# Outcome and clinico-biological characteristics of advanced breast cancer patients with surgically resected brain metastases: a multidisciplinary approach

**DOI:** 10.3332/ecancer.2013.309

**Published:** 2013-04-18

**Authors:** Elisabetta Munzone, Cecilia Casali, Gaetano Aurilio, Edoardo Botteri, Alessandro Perin, Giuliana Pelicci, Paola Brescia, Angela Sciandivasci, Laura Adamoli, Giuseppe Viale, Francesco DiMeco

**Affiliations:** 1 Department of Medicine, Division of Medical Senology, European Institute of Oncology, Milan, Italy; 2 Unità Operativa Neurochirurgia I, Istituto Neurologico Carlo Besta, Milan, Italy; 3 Division of Epidemiology and Biostatistics, European Institute of Oncology, Milan, Italy; 4 Department of Experimental Oncology, European Institute of Oncology, Milan, Italy; 5 Division of Pathology, European Institute of Oncology, Milan, Italy; 6 University of Milan, Milan, Italy

**Keywords:** breast cancer, brain metastases, surgery, medical treatments, survival outcomes

## Abstract

**Background:**

Despite improvements in brain surgery and radiotherapy, patients with brain metastases (BM) from breast cancer still have a poor prognosis. The aim of the present study is to evaluate the outcome of a multimodal therapeutic strategy in an unselected cohort of patients.

**Methods:**

We retrospectively reviewed 24 breast cancer patients who developed BM and were treated with brain surgery, radiotherapy, and/or systemic therapy in the same institutions.

**Results:**

Primary treatment for BM was surgery in the whole cohort, radiotherapy in 11 patients, radiotherapy combined with systemic therapy in nine patients, and systemic therapy as single treatment in six patients (chemo/targeted therapy *n*= 4; hormonal therapy *n*=2). The median time from breast cancer diagnosis to brain surgery was 57.6 months (range 1.8–130.7 months). The overall survival from surgery for BM was 22 months and the overall survival from BM surgery by presence of other metastatic sites at surgery was 25 months for patients with BM only and 11 months for patients with other metastatic sites (*p*=0.046).

**Conclusion:**

Although this study is retrospective and limited by the small number of patients, the overall survival of 22 months from the time of brain surgery represents an excellent outcome. The multidisciplinary approach that combines the efforts of specialists from different disciplines leads to satisfactory results for patients in terms of survival in the current clinical practice and prospective subtype-oriented trials are urgently required in this category of patients.

## Introduction

BM are the most common malignancy of the central nervous system (CNS), and their incidence has dramatically increased in recent years, mainly due to the improvements in systemic chemotherapy. This is particularly valid in BM from breast cancer whose patients have, even with the best available treatments, a significant mortality and morbidity [1].

Approximately 10–15% of patients with breast primary tumours will develop BM during the course of disease [2], reaching 30% when considering autopsy series [3].

Brain relapse typically occurs within two to three years after the removal of the breast tumour [4]

The median survival after CNS metastasis in breast cancer patients is approximately four months, and the one-year survival rate is about 20% [5].

As one of the major types of breast cancer metastasis, BM are of particular interest because of the high mortality resulting from brain lesions and resistance to chemotherapies, mainly due to the inability of many conventional drugs to cross the blood–brain barrier (BBB).

The lack of effective treatments for BM represents an important social health problem, and there is no consensus on the therapeutic strategy, sequence of treatments, and combination therapies, so far.

Several studies have reported some factors associated with the development of BM: young age, lymph nodal status, high tumour grade, and distant metastases. In addition, patients with metastatic triple-negative breast cancer (ER, PR, HER-2 unamplified) and HER-2-overexpressed subtype tend to develop brain metastasis at a higher rate [1, 6].

In recent years, besides the rise of new promising agents and combination therapies, a crucial role in the care of metastatic breast cancer is being played by the multidisciplinary approach in the development of efficacious treatment strategies, characterised by interaction and active cooperation among radiologists, surgeons, pathologists, radiation oncologists and medical oncologists. Unfortunately, in routine clinical practice, metastatic breast cancer cases are discussed much less frequently than early breast cancer cases in multidisciplinary meetings. Therefore, the opportunity for a selected local treatment is not fully exploited [7].

Based on this scenario, we sought to evaluate a consecutive series of 24 patients with breast cancer metastatic to the brain, treated with stereotactic surgery followed by medical treatments, either radiotherapy or systemic treatment, to assess the effect of sequential and combined therapies in terms of survival outcomes.

## Patients and methods

This analysis was retrospectively carried out in 24 breast cancer patients who underwent surgery for BM at Besta Institute (Milano, Italy) and were followed at the European Institute of Oncology, Milan, Italy, for the primary breast tumour.

Clinical records of breast cancer patients with BM were analysed retrospectively. In detail, we collected different clinical features of the patients including age, stage at breast cancer diagnosis, histological tumour characteristics, time interval between the diagnosis and first recurrence, site of first recurrence, number of metastases, treatment modalities for breast cancer (adjuvant and palliative), treatment modalities for BM (surgery, radiotherapy) and systemic therapy thereafter, follow-up interval, and the time period between the diagnosis and death.

We excluded patients with a second primary malignancy other than breast cancer. Diagnosis of breast cancer was histologically confirmed.

All patients provided a signed informed consent for the use of their clinical, surgical, and pathological data.

Data on patients’ medical history, concurrent diseases, type of surgery, and pathological assessment of morphological and biological features were combined.

Primary tumours were classified according to the World Health Organization Histological Classification of Breast Tumors, as modified by Rosen and Obermann [8]. Tumour grading was assessed according to Elston and Ellis [9].

Immunostaining for the localisation of ER and PgR, HER-2 protein, and Ki-67 antigen was performed on consecutive tissue sections according to the standard procedures.

Only nuclear reactivity was taken into account for ER, PgR, and Ki-67 antigen, whereas only an intense and complete membrane staining in >10% of the tumour cells qualified for HER-2 overexpression (3+). For this analysis, HER-2 scores of 0 and 1+ were considered negative. FISH assays (using the PathVysion HER-2 DNA kit, Vysis–Abbott, Des Plaines, IL) were performed in cases with equivocal (2+) immunohistochemical results to identify cases with gene amplification (HER-2 to chromosome 17 centromere ratio ≥2).

The results for ER, PgR and Ki-67 were recorded as the percentage of immunoreactive cells observed among at least 2,000 neoplastic cells.

Patients were categorised in five categories based on the tumour biology: Luminal A with ER and PgR >1%, G1/G2, HER-2 negative, Ki67 low (<14%); Luminal B with ER and/or PgR >1%, G3, HER-2 negative, Ki67 high (>14%); Luminal B (HER-2 positive) with ER and/or PgR >1%, G any, HER-2 positive, Ki67 any; HER-2 positive (non-luminal) with HER-2 overexpressed or FISH amplified and ER and PgR absent; triple negative with both ER and PgR 0%, and HER-2 not overexpressed or FISH not amplified.

Clinical and pathological data were entered by medical oncologists into a ‘user-friendly’ database designed with Microsoft Access®, and checked by a data manager and a quality data reviewer.

## Statistical analyses

Median time from primary surgery to BM was calculated in the group of patients who were free of BM at primary diagnosis, and the log-rank test was used to evaluate differences among subgroups of patients.

The Wilcoxon Signed Rank Test was used to evaluate differences in ER and PgR expression from the primary tumour to the metastatic tissue in the brain.

Overall survival was defined as the time interval from brain surgery to death from any cause, or last date of follow-up, and plotted using the Kaplan-Meier method. The Log-rank test was used to test survival differences among subgroups of patients. A multivariate Cox proportional hazards regression model was used to evaluate the independent effect of the characteristics, the results of which were significant in unvaried analysis.

All analyses were performed with SAS software (SAS Institute, Cary, NC) and R software (The R Development Core Team 2004; Free Software Foundation, Boston, MA).

## Results

### Patient characteristics

This analysis included 24 patients with breast cancer metastatic to the brain who underwent surgery for BM between 2006 and 2011.

Patient characteristics are summarised in Table 1.

Upon primary breast cancer diagnosis, 23 out of 24 (96%) patients were metastases free and most of the patients (54.2%) were between 35 and 49 years of age.

Immunohistochemical classification was available for 23 pts: Luminal A tumours were not represented; there were 43.5% of pts with Luminal B HER-2 negative tumours, and 43.4% pts with either HER-2 positive (30.4%) or triple-negative (13%) tumours.

Twenty patients received adjuvant treatment, of whom 11 patients (55%) received anthracycline-based chemotherapy, two patients (10%) taxane and anthracycline combined chemotherapy, five patients (25%) trastuzumab combined chemotherapy, one patient (5%) endocrine therapy alone and nine (45%) after chemotherapy, two patients (10%) chemotherapy with CMF regimen and only one (5%) paclitaxel as monotherapy.

The brain was the first and solitary site of metastases in eight patients (42%). Three patients (12%) presented with multiple sites in the brain district, while 11 patients (46%) developed initial relapse in other sites (no brain), including bone (17%), lymph nodes (21%), breast (4%) and mediastinum (4%); data for two patients were missing.

All patients were submitted to stereotactic brain surgery for intracranial disease, mainly for solitary and well-identified lesions. In only three patients, multiple lesions in different sites were detected and then removed. Surgical resections were not radical in ten patients, while missing data were provided for three patients. Consequently, ten patients received optimal surgery with margins free of disease.

The median time from breast cancer diagnosis to brain surgery was 57.6 months (range 1.8–130.7 months). Patients with HER-2 positive tumours had a median time to brain surgery of 28 months, patients with Luminal B HER-2 positive 34 months, patients with triple-negative tumours 42 months and Luminal B HER-2 negative 68 months.

### Survival analysis

**Figure 1:**
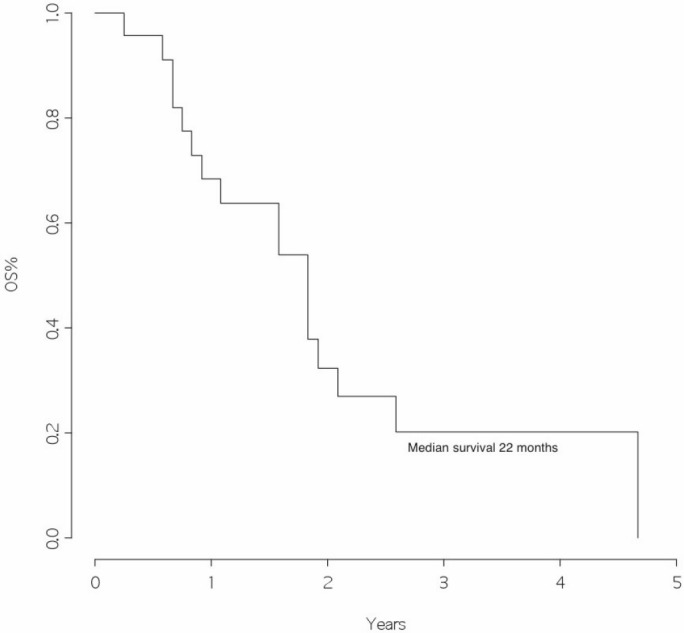
Overall survival from surgery for brain metastasis

**Figure 2:**
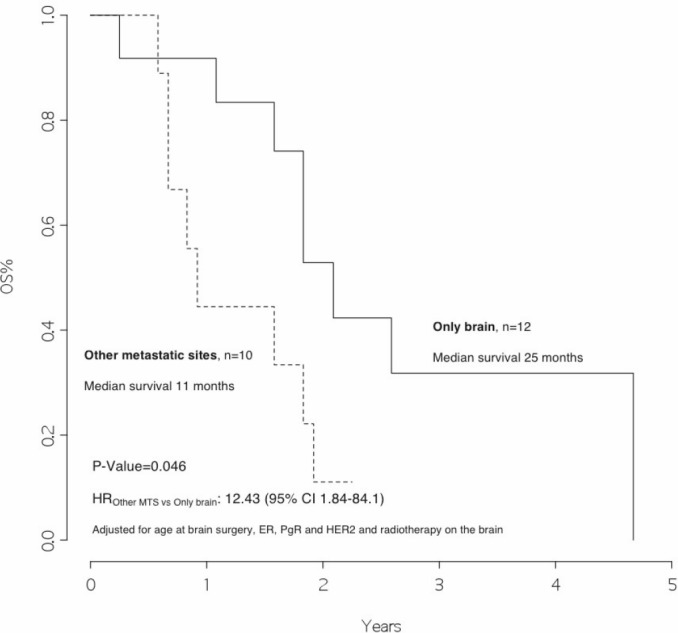
Overall survival from surgery for brain metastasis, by presence of other metastatic sites at surgery

The overall survival from surgery for BM was 22 months (Figure 1), and the overall survival from BM surgery by the presence of other metastatic sites at surgery was 25 months for patients with BM only and 11 months for patients with other metastatic sites, *p*=0.046 (Figure 2) with a hazard ratio (HR)_Other MTS versus Only brain_: 12.43 (95% CI 1.84–84.1).

The multivariable analysis (Table 3) showed that patients with other metastatic sites other than brain, and ER/PgR negative, had a significantly worse outcome as compared with others, HR 14.8 (95% CI 2.55–85.6, *p *value 0.003) and 26.2 (95% CI 2.45–278.8, *p *value 0.007), respectively. Patients with HER-2 overexpressed had a better outcome as compared with the others, HR 0.08 (95% CI 0.01–0.73, *p *value 0.025).

### Treatment of patients after brain surgery

Whole brain radiotherapy (or cyberknife) was performed in 11 patients (46%) as treatment after surgery, when multiple metastases were diagnosed or non-radical brain surgery was performed. Radiotherapy treatment was not indicated in nine patients (37%), while data were missing for four patients (17%).

Patients who received radiotherapy had a median overall survival of 23 months as compared with those who did not, with a median overall survival of 11 months, *p* =0.020, HR_RT versus no RT_: 0.21 (95% CI 0.04–1.16) (Figure 4).

With regard to medical treatments, seven patients received trastuzumab plus chemotherapy, mainly oral drugs (such as methotrexate and cyclophosphamide), five patients were treated with chemotherapy alone (both intravenous and oral combinations), three patients received hormonal drugs orally (letrozole and exemestane), one patient was treated with intracranial chemotherapy, no therapy was given for medical reasons in four patients, and data was missing for four patients.

### Receptor discordance between primary breast cancer and brain metastases

Quantitative changes in ER, PR and HER-2 immunoreactivity between primary breast cancer and BM were evaluated in 14 of 23 patients for ER and PR (no data were available for ten patients), and in 16 of 23 patients for HER-2 (no data for comparison in seven patients). One patient presented diagnosis of occult breast carcinoma and therefore immunohistochemistry (IHC) was not assessed.

We had information on immunohistochemical status on matched primary tumours and BM in 14 pts for ER and PgR, and in 16 pts for HER-2. The comparison between IHC status in primary and in brain metastatic tissue is shown in Table 2.

**Figure 3:**
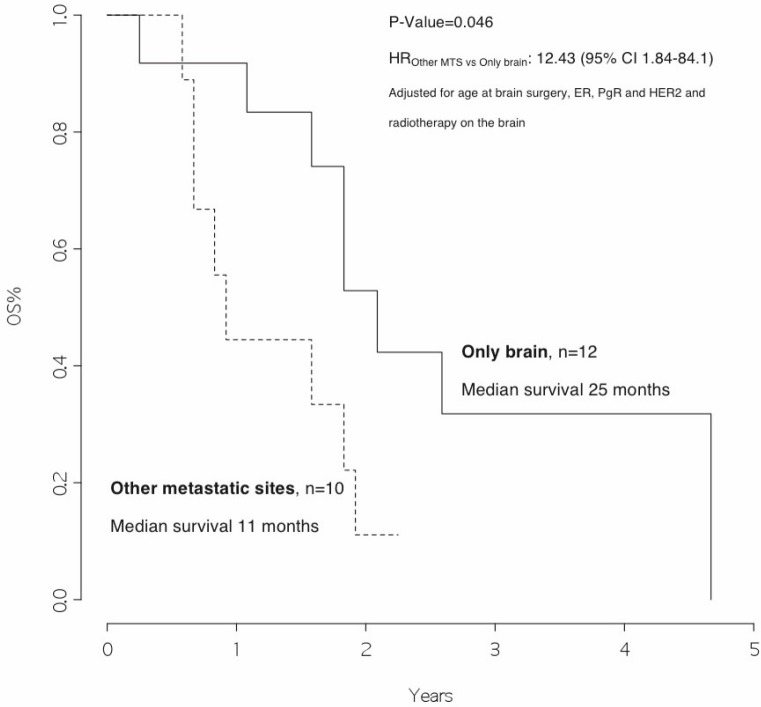
Overall survival from surgery for brain metastasis, by presence of other metastatic sites at surgery

**Figure 4:**
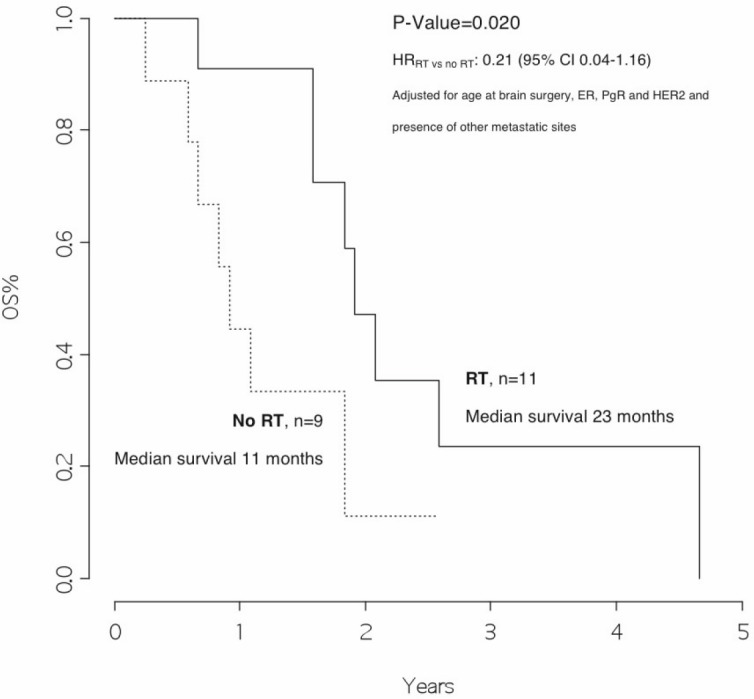
Overall survival from surgery for brain metastasis, by radiotherapy on the brain

None of the pts changed HER-2 status, but 3 pts with ER positive in the primary tumour shifted to ER negative in the BM, 4 pts with PgR positive shifted to PgR negative in the BM and 1 pt with PgR negative in the PT showed PgR positive in the BM (Figure 3).

## Discussion

This retrospective study was conducted in a group of patients followed for the primary tumour and operated for BM in the same institutions. Although this study is restricted to a few patients, we found an overall survival of 22 months from the time of brain surgery. This represents an excellent outcome when compared with data from the literature, where a median survival of approximately four months is reported after the diagnosis of BM [5].

All patients included in this retrospective study were selected for eligibility for surgery, because of a limited brain disease, and almost all have received systemic therapy after surgery, which included both chemotherapy and radiotherapy. This is not a negligible fact and might certainly influence the results. However, this evidence confirms that, where possible, the multidisciplinary approach that combines the efforts of specialists from different disciplines leads to satisfactory results for patients in terms of survival in the current clinical practice.

In addition, we observed that patients with disease limited only to the brain had a significantly better survival rate than patients who also had other metastatic sites (25 versus 11 months, *p *value 0.046). Moreover, patients who received radiotherapy after surgery had a significantly better outcome as compared with those who did not (23 versus 11 months, *p* value=0.020, HR_RT versus no RT_: 0.21 [95% CI 0.04–1.16]).

These results are in line with evidence from randomised clinical trials conducted in recent years, indicating that radiotherapy could reduce the intracranial disease relapse rate from 70% to 30–40% [10, 11].

Analysing the results for breast cancer subtypes, the majority were represented by patients with triple-negative tumours or with HER-2 overexpression (56.4%), the remaining were Luminal B/HER-2 negative (43.6%) and there were no patients with Luminal A cancer.

These data are consistent with the findings in literature, where the majority of patients who develop brain metastases have a primary triple-negative or HER-2 positive tumour [1, 6].

Nonetheless, in the multivariable analysis of our series of patients, those with HER-2 overexpressed had a better outcome as compared with the others, HR 0.08 (95% CI: 0.01–0.73, *p* value 0.025).

One possible explanation for this finding might be that patients with HER-2 positive breast cancer after BM under trastuzumab treatment have a prolonged survival because of the successful clinical control of extracranial disease related to target treatment [12].

Another interesting aspect emerging from this study is the possible changes in the biology of the tumour by comparing the primary tumour with brain metastases. There is rising evidence that tumour receptor status may change dynamically during the natural history of the disease.

Particularly, comparing the immunohistochemical data, we observed that none of the patients had a change of the HER-2 status, while three and four patients, respectively, lost the expression of ER or PgR from primary tumour to brain metastasis. The modification of about 20% in the expression of hormone receptors, particularly ER, is compatible with the literature data from other studies where changes in the receptor expression from the primary tumour to metastases were studied [13, 14].

These data should be considered, especially when a personalised treatment is chosen for the patient.

In conclusion, although there are some limitations in the present study, such as the retrospective design, the single-institution character and the limited number of patients, together with some missing data in the biological profile of brain metastases, we strongly believe that subtype-oriented prospective trials are urgently required to further exploit the importance of a combined and multidisciplinary approach in this category of patients.

## Figures and Tables

**Table 1.** Characteristics of patients and association with time from surgery

Variable	Classification	All patients *n*=24	Median time from surgery	*p* value
**Age**	<35	7 (29.2)	68.0	0.31
	35–49	13 (54.2)	51.5	
	≥50	4 (16.7)	29.0	
**ER**	Negative	10 (43.5)	30.5	0.03
	Positive	13 (56.5)	66.0	
**PgR**	Negative	11 (47.8)	30.5	0.03
	Positive	12 (52.2)	66.0	
**HER-2/Neu**	Overexpressed	10 (43.5)	32.5	0.20
	Not overexpressed	13 (56.5)	66.0	
**Ki-67**	<14	–	–	0.03
	14–35	10 (45.5)	32.0	
	≥35	12 (54.5)	71.5	
**Molecular subtype**	Luminal A	–	–	0.14
	Luminal B HER-2-	10 (43.5)	68.0	
	Luminal B HER-2+	3 (13.0)	34.0	
	HER-2+	7 (30.4)	28.0	
	Triple negative	3 (13.0)	42.0	

Information on tumour characteristics was not available for all patients. ER, estrogen receptor; PgR, progesterone receptor; HER-2+: HER-2/Neu overexpressed; HER-2−: HER-2/Neu not overexpressed

**Table 2.** Comparing ER, PgR and HER-2/Neu status in the primary and metastatic tissue

Brain metastasis									
		**ER−**	**ER+**		**PgR−**	**PgR+**		**HER-2/Neu−**	**HER-2/Neu+**
**Primary**	**ER−**	7	0	**PgR−**	7	1	**HER-2/Neu −**	10	0
	**ER+**	3	4	**PgR+**	4	2	**HER-2/Neu +**	0	6

ER, estrogen receptor; PgR, progesterone receptor; HER-2+: HER-2/Neu overexpressed; HER-2−: HER-2/Neu not overexpressed

**Table 3.** Overall survival from surgery for brain metastasis: multivariable analysis

Variables	Comparison	HR	95% CI inf	95% CI sup	*p* value
**Age at brain surgery**	Continuous	1.09	0.99	1.12	0.061
**Metastatic site other than brain**	Yes versus No	14.8	2.55	85.6	0.003
**ER/PgR**	Both negative versus others	26.2	2.45	278.8	0.007
**HER-2**	Overexpressed versus not overexpressed	0.08	0.01	0.73	0.025
